# Platelet transcriptome profiles provide potential therapeutic targets for elderly acute myelocytic leukemia patients

**DOI:** 10.1186/s12967-021-03041-8

**Published:** 2021-09-10

**Authors:** Jizhang Bao, Xinhua Zhao, Jiahui Lu, Zhaoyang Hu, Minghui Hu, Xiaoxia Hu, Libing Wang, Qi Hu, Weiling Sun, Jie Wang, Hailin Chen, Hao Lu, Changgui Li, Jing Xu, Yongming Zhou, Wenwei Zhu

**Affiliations:** 1 The Hematological Dept., Yueyang Hospital of Integrated Traditional Chinese and Western Medicine, Shanghai University of Traditional Chinese Medicine, Shanghai, China; 2 Basic Medical College, Shanghai University of Traditional Chinese Medicine, Shanghai, China; 3 The Hematological Dept., Shanghai Municipal Hospital of Traditional Chinese Medicine, Shanghai University of Traditional Chinese Medicine, Shanghai, China; 4 Fun-Med Pharmaceutical Technology (Shanghai) Co., Ltd., RM. 501, 1188 Jiangyue Road, Minhang District, Shanghai, China; 5 Department of Hematology, Changhai Hospital, Shanghai, China

**Keywords:** Acute myeloid leukemia, Platelet RNA sequencing, Mitogen activated protein kinase, Activating transcription factor 4

## Abstract

**Background:**

Acute myeloid leukemia (AML) is the most common acute leukemia in adults, with a median age of 68 in clinical diagnosis. About 60% patients are over 60 years old. There are various treatment options for AML patients. But for elderly patients, the complete remission rates are disappointing due to genetic, molecular, and age-related factors. Development of next-generation sequencing technologies makes it possible to seek individual strategies for patients in different ages. This study analyzed transcriptome profiles in platelets of AML patients in different ages for the first time.

**Methods:**

Platelet RNA sequencing in AML of ten elderly and seven young patients were performed with Illumina TruSeq Stranded mRNA library Prep Kit and Illumina HiSeq4000 sequencing instrument. With the FASTQ sequencing data obtained, statistical analyses between elderly with young AML patients were analyzed by R program. GO and KEGG enrichment analyses were performed via R package clusterProfiler. TOP 10 down-regulated/up-regulated genes in elderly patients compared to young patients were selected with the threshold of |L2FC| > 2 and padj ≤ 0.0001. The down-regulated gene ATF4 was chosen by GSEA analysis and ROC analysis with AUC > 0.95.

**Results:**

We found 3059 genes with differential transcript levels (GDTLs) in AML patients of different age. Among them, 2048 genes are down-regulated and 651 genes are up-regulated in elderly patients. We found that gene transcript profiles in elderly patients is obviously different from those in young patients, including a collection of down-regulated genes related to proteins processing in endoplasmic reticulum and immunity. We further identified that genes of pathway in cancer and mitogen activated protein kinase (MAPK) pathway, involved in natural immunity and metabolism, are significantly down-regulated in elderly patients. Among all screened genes with decreased transcript levels, we believe that activating transcription factor 4 (ATF4) is a biomarker indicating different chemotherapy strategies for elderly patients.

**Conclusions:**

In summary, gene transcript profiles are different in platelets of elderly and young AML patients. And ATF4 can be a useful biomarker indicating different chemotherapy strategies for AML patients with different ages.

**Supplementary Information:**

The online version contains supplementary material available at 10.1186/s12967-021-03041-8.

## Highlights

1. Gene transcript profiles in the elderly AML patients are different from those in young patients.
2. Statistical analysis reveals the down-regulation of protein processing in ER and pathways related to immunity in elderly patients.
3. GEO and GSEA analyses demonstrate the genes of cancer pathway and mitogen activated protein kinase (MAPK) pathway, involved in natural immunity and metabolism, are largely down-regulated in elderly patients.
4. ATF4 is a biomarker indicating different therapeutic strategies for elderly patients.

**Supplementary Information:**

The online version contains supplementary material available at 10.1186/s12967-021-03041-8.

## Introduction

Acute myeloid leukemia (AML), as the most common acute leukemia in adults, is a malignant cancer characterized by abnormally differentiated and proliferated hematopoietic stem cells infiltrating bone marrow, blood and other tissues [1]. The general therapeutic strategies for AML patients changed little for many years, with a combination of consolidation therapy of hematopoietic-cell transplantation and conventional chemotherapy following cytarabine or anthracycline induction for complete remission [2, 3]. Several problems arise since the median age of AML patients is 68 years old [4]. Current clinical data show a cure rate of 35% to 40% in AML patients who are 60 years of age or younger, but less than 15% in patients over 60 years old [2, 4]. Along with high risk of relapse, the approximate survival of elderly patients is only 5 to 10 months.

The heterogeneity of AML accounts for the generally low cure rate for patients. Specially, a collection of various adverse prognostic factors and comorbidities of elderly patients is the determining limitation in chemotherapy and hematopoietic-cell transplantation [5]. Although a study reported autologous hematopoietic stem cell transplantation (HCT) to be a considerable strategy with distinct anti-leukemic effectiveness for elderly patients, the outcome of elderly patients is still disappointing [6]. In recent years, the development of RNA sequencing over microarray has made it possible to analyze the karyotype of AML patients, thereby providing different treatment measures to patients with different subtypes [7–9]. There are several studies analyzing the genes with differential transcript levels of AML patients. Genes such as *DNMT3A*, *NPM1* and *CEBP1* are proved to be alternatively regulated in AML patents [10–12]. These biomarkers contribute to a better understanding of AML and make great sense in diagnosis and prognosis for patients [13].

With the development of technologies, there is a better understanding for important roles of transcripts in platelets in pathophysiology [14]. Although many studies have analyzed the changes in the transcript profiles of AML patients, studies of platelet RNA sequencing on AML patients are limited. Besides, the differences in gene transcript profiles between elderly and young AML patients have yet to be analyzed.

In this study, we analyzed the difference in gene transcript profiles between young and elderly patients. We obtained 17 blood samples from AML patients in Shanghai Yueyang Integrated Traditional Chinese Medicine and Western Medicine Hospital affiliated to Shanghai University of Traditional Chinese Medicine, and characterized them to two different groups according to age. 10 samples from AML patients over 60 years old are classified to elderly group, and 7 samples left belong to young group. After RNA sequencing in platelets, the following statistical analysis identifies 3059 genes with differential transcript levels in AML patients of different age, with 2408 genes down-regulated and 651 genes up-regulated in elderly patients, suggesting that gene transcript profiles in elderly patients is obviously different from that in young patients. Besides the global down-regulation gene profile, gene transcript profiles related to protein processing in endoplasmic reticulum and immunity are notably impaired in elderly patients, which explains the disappointing outcome of elderly AML patients.

We further reveal that genes of pathway in cancer and mitogen activated protein kinase (MAPK) pathway, involved in natural immunity and metabolism, are significantly down-regulated in elderly patients. Combined with the top ten down-regulated genes selected with the threshold of |Log2FC| > 2 and padj ≤ 0.0001, we identify the down-regulated gene, activating transcription factor 4 (ATF4) is a biomarker indicating potential therapeutic strategies for elderly patients.

## Methods

### Sample collection and purification

Blood samples of eleven elderly and eight young AML patients were collected from AML patients in Shanghai Yueyang Integrated Traditional Chinese Medicine and Western Medicine Hospital affiliated to Shanghai University of Traditional Chinese Medicine from February 7 2018 to March 14 2019. In accordance with Declaration of Helsinki, this study was approved by the Ethical Committee of the Yueyang Hospital. And written informed consent was obtained from all subjects and/or guardians for the use of their blood samples.

### RNA extraction qualification

Platelets isolations were performed as reported [14]. For RNA sequencing analysis, total RNA was purified with TRIzol reagent (Invitrogen) treatment. After RNA extraction, The RNA quality was measured by the following ways: (a) Agarose gel electrophoresis for integrity and DNA pollution of the RNA; (b) NanoPhotometer spectrophotometer for the RNA purity (the results of OD260/280 and OD260/230); (c) Qubit2.0 Fluorometer for the exact RNA concentration; and (d) Agilent 2100 bioanalyzer for quantitative analysis of RNA integrity.

### Library construction and sequencing

Total platelet RNA of each sample was extracted using RNeasy Micro Kit (Qlagen, cat. no. 74004) per the manufacturer’s instructions. The total RNA was quantified and qualified by Agilent 2100 Bioanalyzer, NanoDrop (Thermo Scientific) and 1% agrose gel. Reverse transcription was achieved with 1 ng of total RNA, oligo (dT) primer and Superscript II reverse transcriptase. To have sufficient platelet cDNA for robust RNA-seq library preparation, each sample was subjected to 20-cycle enrichment PCR, using the KAPA HiFi HotStart ReadyMix PCR kit (Kapa Biosystems, USA), yielding ~ 200 ng of cDNA. The cDNA fragments around 200 bp were then purified with AMPure XP beads, which were amplified by PCR. The purified cDNA library was purified with AMPure XP beads. Then, Qubit2.0 Fluorometer was used to measure the DNA concentration and library dilution, and Agilent 2100 bioanalyzer was used to test the insert size of the library. And qRT-PCR was performed to test the library effective concentration precisely (> 2 nM effective concentration). Next paired-end sequencing was performed by the Illumina HiSeq4000 sequencing instrument, according to the manufacturer’s instructions and as reported [15].

### Statistical analyses

Quality control was performed after the FASTQ sequencing data obtained with fastp software (https://github.com/OpenGene/fastp) to remove over-short, low-quality, undetected and also the adaptor reads. Mapping step was performed with STAR software, an ultrafast universal RNA-seq aligner, using MMP (Maximal Mappable Prefix) method to align the RNA-seq data onto the hg19 reference genome [16]. HTSeq (https://pypi.org/project/HTSeq/) was then used for gene transcript quantification. Gene transcript profiles were analyzed by R program and related packages. Pathway analyses were performed with Gene Ontology (GO) (http://www.geneontology.org) and Kyoto Encyclopedia of Genes and Genomes (KEGG) pathway database (http://www.genome.jp/kegg/). Venn diagram of down-regulated genes in elderly patients compared to existing GSE13888 database was obtained by the interactive Venn diagram viewer jvenn (http://jvenn.toulouse.inra.fr/app/index.html) [17]. Statistical significance of data was assessed by one-way ANOVA test.

## Results

### Comparison of gene transcript profiles between young and elderly AML patients

**Fig. 1:**
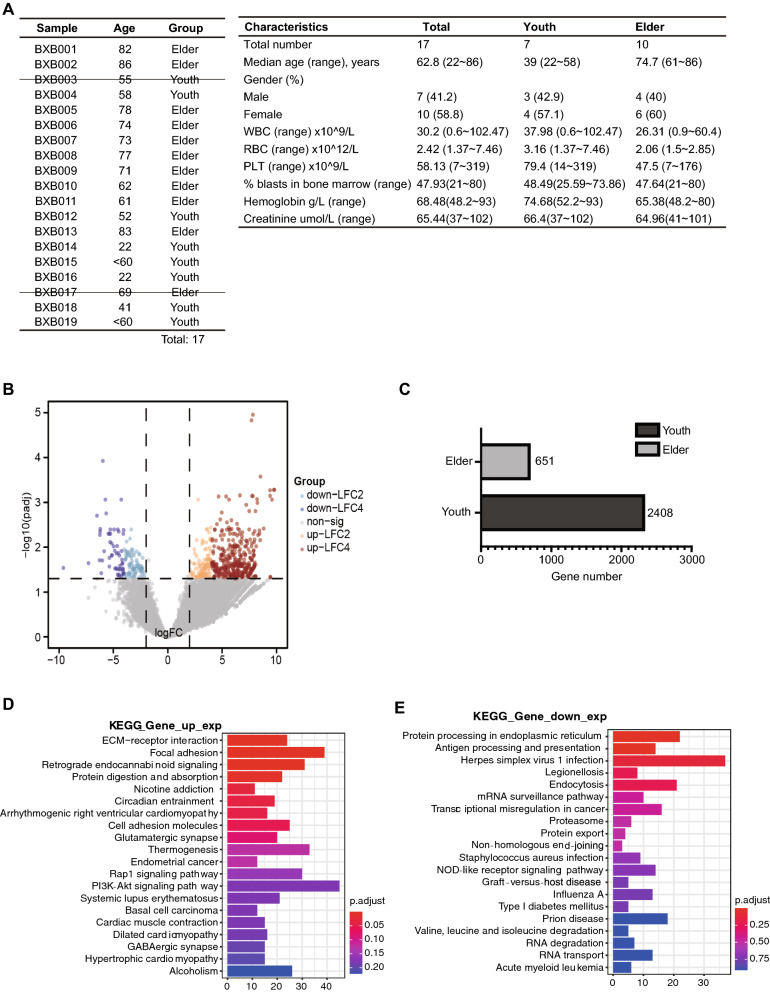
Gene transcript profiles of young and elderly group. **A** Basic information of clinical samples. **B** Volcano plot of young group compared to elderly group. Up-regulated genes are marked with blue and purple, and down-regulated genes are marked with orange and brown. **C** Total GDTLs between young and elderly group. **D** KEGG analysis of up-regulated genes in elderly group. **E** KEGG analysis of down-regulated genes in elderly group

In this study, we collected 19 blood samples from AML patients admitted to Shanghai Yueyang Integrated Traditional Chinese Medicine and Western Medicine Hospital affiliated to Shanghai University of Traditional Chinese Medicine, and characterized them to two different groups according to age (Fig. 1A). AML patients over 60 years old are classified to elderly group, the others belong to young group [18]. Following platelets and RNA isolation, we performed platelet RNA sequencing and obtained the raw FASTQ data. After scanning, probe-level data were preprocessed, including background correction, quantile normalization and summarization (Additional file 1: Table S1). Sample AML003 was excluded due to the low quality of RNA sequencing data. The RNA sequencing data of sample AML017 was excluded for following analyses as requested by this patient. Thus, we got 10 sequencing results for elderly patients and 7 for the young.

By analyzing the expression data of the two groups with R program, we screened GDTLs between elderly and young group with the threshold of |Log2FC| > 2 and P_value ≤ 0.05. As shown in the volcano plot, a total of 3059 GDTLs were screened, among which 651 (21.28%) genes were up-regulated and 2408 (78.72%) genes were down-regulated in the elderly group compared to the young group, suggesting a primary downward trend of gene transcript profile in elderly AML patients (Fig. 1B, C, Additional file 2: Table S2).

To further understand conversation and variation of GDTLs at the level of organisms between elderly and young group, we performed KEGG pathway enrichment analyses to functionally annotate GDTLs via the R package clusterProfiler (Additional file 3: Table S3). KEGG is a database resource that integrates genomic, chemical and systemic functional information [19]. Results showed that up-regulated genes in elderly group were mainly enriched in pathways such as ECM-receptor interaction, focal adhesion and retrograde endocannabinoid signaling, and down-regulated genes were mainly enriched related to proteins processing in endoplasmic reticulum and immunity (Fig. 1D, E),

### Protein–protein interactions (PPI) and GO analysis of down-regulated genes in elderly group

**Fig. 2:**
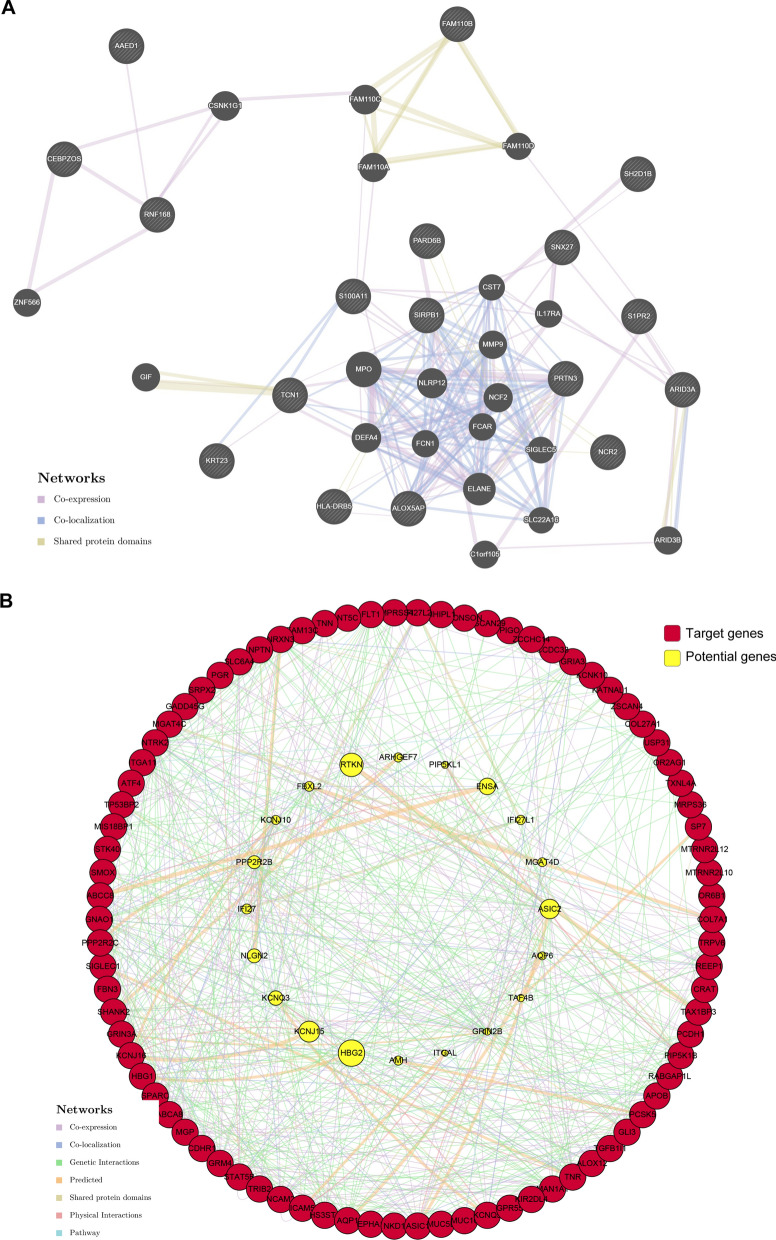
PPI networks of GDTLs in elderly group. **A** PPI network of up-regulated genes in elderly group with the threshold of |L2FC| > 2 and padj ≤ 0.0001. **B** PPI network of down-regulated genes in elderly group with the threshold of |L2FC| > 2 and padj ≤ 0.0001

Given that gene transcript profiles in the elderly group were mainly down-regulated, we speculated down-regulated genes were of great importance for different outcomes in AML patients of different ages. To construct a protein–protein interactions (PPI) network, we analyzed up-regulated genes in elderly group with the threshold of |Log2FC| > 2 and padj ≤ 0.00001, and found that they co-expressed or co-localized but rarely involved in a common pathway (Fig. 2A). On the contrary, the down-regulated genes selected with the same criterions had more tight connections in genetic and physical pathways (Fig. 2B). The difference reminds us that down-regulated genes in elderly group compared to the young group could be the biomarkers indicating different therapeutic strategies in clinic.

**Fig. 3:**
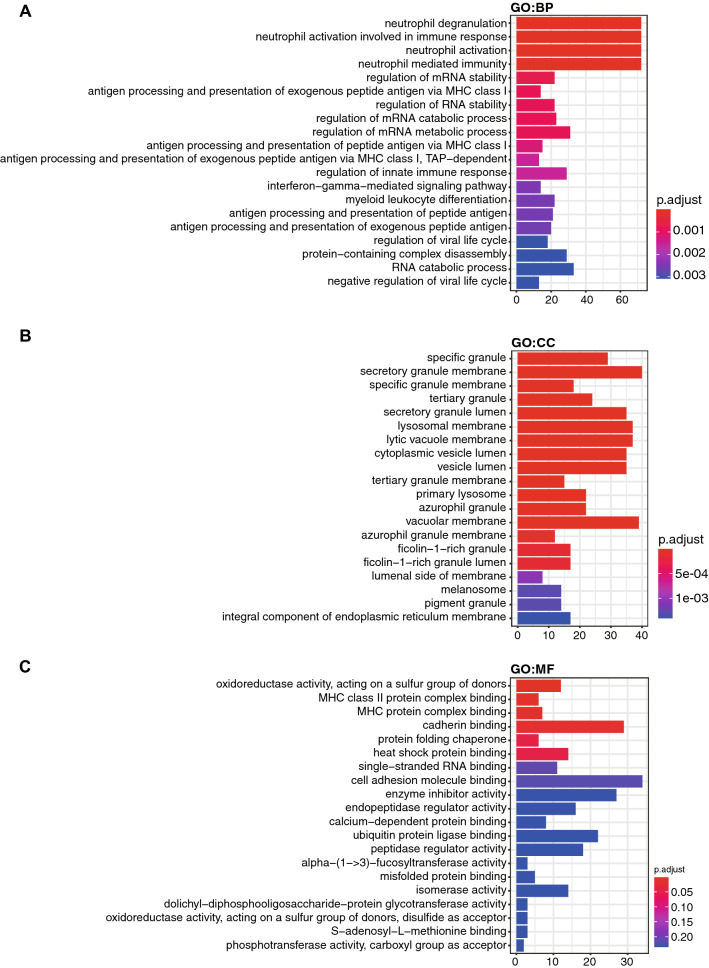
GO annotations of down-regulated genes in elderly group with the threshold of |L2FC| > 2 and padj ≤ 0.0001. **A** Biological process. **B** Cellular component. **C** Molecular function

We next performed GO analysis to further annotate the biological roles of down-regulated genes, including biological process (BP), cellular component (CC) and molecular function (MF) (Additional file 4: Table S4). The top 4 GO terms related to biological process of the down-regulated genes in elderly group were neutrophil degranulation, neutrophil activation involved in immune response, neutrophil activation and neutrophil mediated immunity (Fig. 3A). In line with KEGG analyses, since AML patients suffer hematopoietic stem cells differentiating and proliferating abnormally, which impairs the immune system, the RNA sequencing based on platelets in this study is quite reliable. Meanwhile, the top 4 cellular component terms related to pathways of granule metabolism (Fig. 3B). Notably, the top molecular function term was oxidoreductase activity (Fig. 3C), suggesting that certain pathways of metabolism changed greatly in elderly patients.

### ATF4 and STAT5B are biomarkers indicating potential therapeutic strategies for elderly patients

**Fig. 4:**
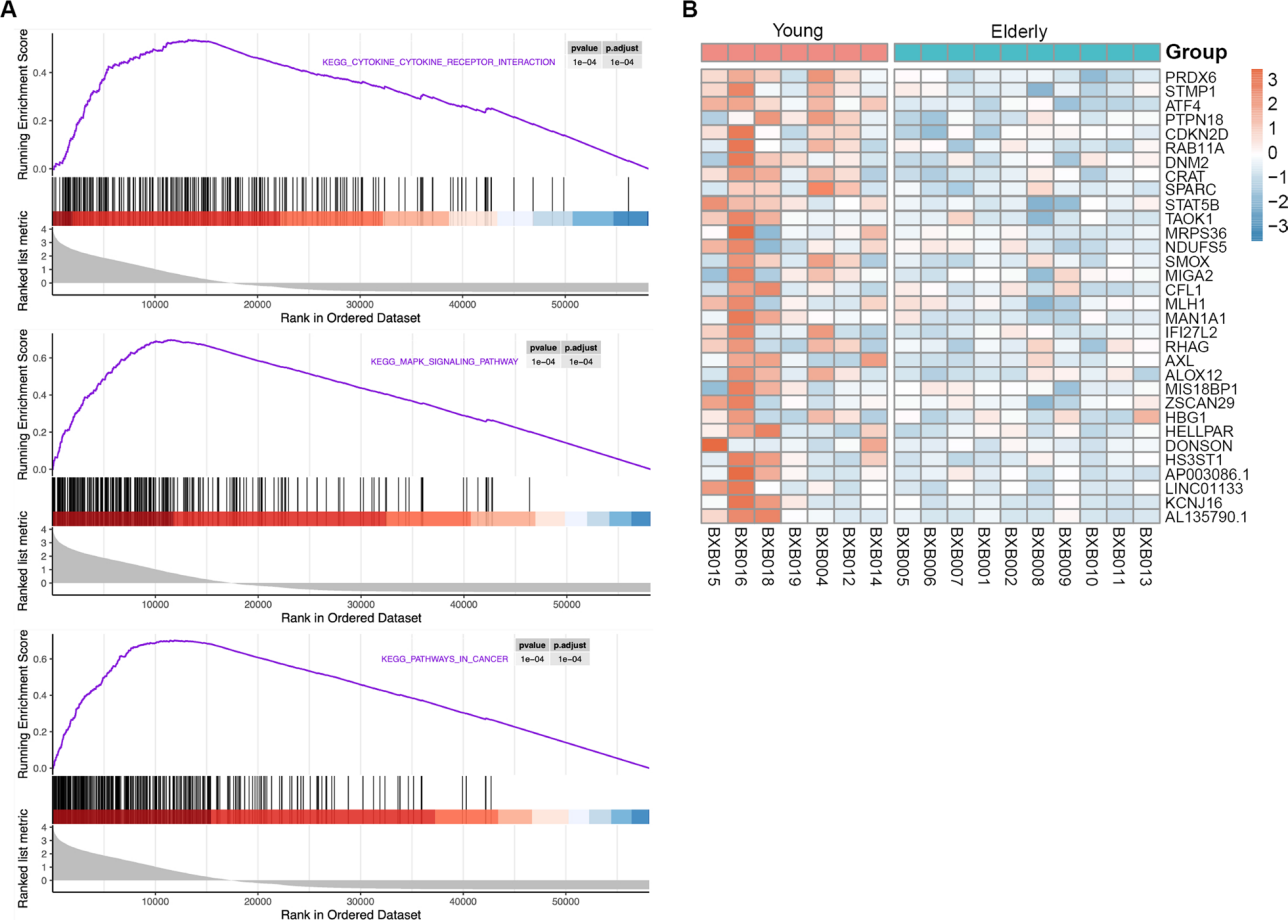
GESA with down-regulated gene sets in elderly group. **A** The top 3 pathways in which the down-regulated genes enrich. **B** Heatmap of down-regulated genes

Gene Set Enrichment Analysis (GSEA) was applied subsequently to extract biological insights into these down-regulated genes sharing common biological processes [20]. Based on GSEA, there were 183 down-regulated mixing pathways in the elderly group (Additional file 5: Table S5). We noticed that the top 3 terms were cytokine-cytokine receptor interaction, MAPK signaling pathway and pathways in cancer (Fig. 4A). MAPK pathway regulates various cellular processes including proliferation and differentiation [21], which suggests the genes involved in MAPK pathway may be of significance for AML therapy.

**Fig. 5:**
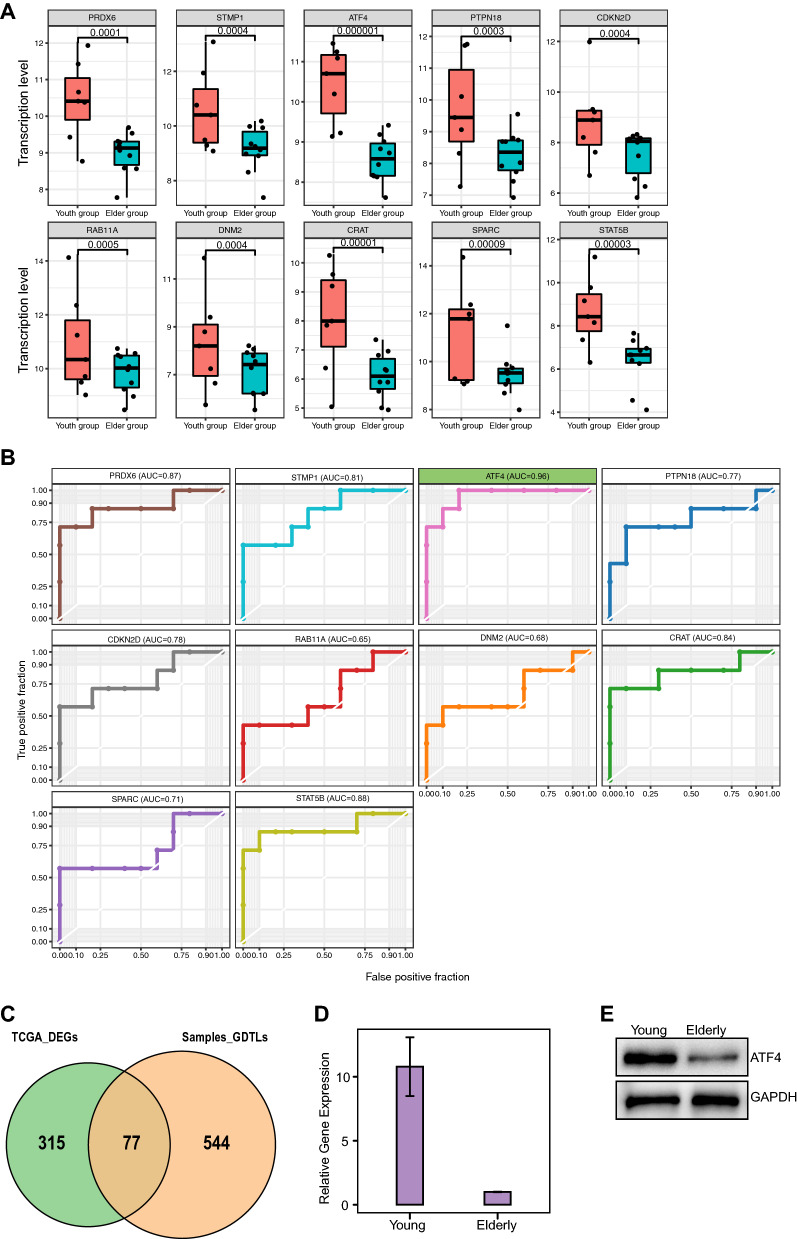
ROC curves of selected 10 down-regulated genes. **A** Gene variations between elderly and young group. **B** ROC analysis. **C** Venn plot of DEGs of young and elderly patients from TCGA AML dataset compared to GDTLs of young and elderly patients in this study, with the threshold of |L2FC| > 1 and padj ≤ 0.05. **D** qPCR of platelets in young and elderly AML patients. **E** Western blotting analysis of platelets in young and elderly AML patients

To identify worthwhile genes, we screened 32 genes down-regulated in the elderly group with the threshold of |Log2FC| > 2 and padj ≤ 0.00001, with the heatmap plot showing obvious differential transcript levels (Fig. 4B). We further selected the top 10 genes, PRDX6, STMP1, ATF4, PTPN18, CDKN2D, RAB11A, DNM2, CRAT, SPARC, STAT5B, compared the expression levels and confirmed the variations between elderly and young group (Fig. 5A). Through a cross-comparison of the gene sets in MAPK pathway with these 10 genes, we found ATF4 could be a potential biomarker indicating the different outcomes between elderly and young AML patients. To confirm our speculation, we performed ROC analysis and found that the AUC score of ATF4 is extremely high (AUG = 0.95), indicating that ATF4 is more reliable to be a biomarker than other 9 genes (Fig. 5B). We further screened 394 different expressing genes between young and elderly AML samples from TCAG database (https://portal.gdc.cancer.gov) with |log2fc| > 1 and padj ≤ 0.05, and compared the result with our study. Unexpectedly, even though there were 77 genes screened coincident with our results, ATF4 is not among them (Fig. 5C). To confirm our RNA-sequencing results, we performed qPCR as well as western blotting analysis to detect mRNA and protein levels in young and old AML patients. These results revealed that the expression level of ATF4 is much higher in young patients compared to the elderly (Fig. 5D, E). Since up-regulation of ATF4 was reported to promote hematopoietic stem cells (HSCs) survival, we thus identified the transcription factor ATF4 to be an important biomarker guiding the different strategies for AML therapy in elderly patients.

## Discussion

Despite the rapid development of technologies over the past few decades, the therapeutic strategies for AML patients changed little [22]. And there are many analyses in molecular level classifying AML to certain subtypes, but no widely accepted algorithm provides therapeutic guidelines for elderly patients. In this study, using a combination of platelet RNA sequencing and bioinformatic analyses, we analyzed the GDTLs between elderly and young AML patients for the first time, and find a global down-regulation pattern in elder AML patients.

As the anucleate cells, platelets used to be considered irrelevant to transcription until studies detected RNAs in platelets [23]. Transcripts in platelets thus became the resourceful measures in blood-based diagnostic tests [24]. RNA-Seq of tumor-educated platelets (TEPs) has enabled blood-based cancer diagnostics and indicate potential clinical therapies [25]. We sequenced the platelets RNA for the blood cancer, AML. By analyzing transcripts, we confirm the fact that the biological pathways related to immunity in elderly patients were quite different from young patients. Further GSEA analysis reveals that down-regulated gene sets of elderly patients are enriched in MAPK pathway.

Taking into account that MAPK pathway plays an important role in cell proliferation and differentiation as mentioned, we believe that MAPK pathway contributes to the pathological process in AML [21]. With the extensive studies of MAPK cascades, MAPK pathway is believed to take part in cancer development and progression [26]. And drugs based on MAPK pathway have been used in cancer therapies [27]. Combined with our analysis results, we identified a transcription factor ATF4, is down-regulated markedly in elderly patients compared to the young.

ATF4 is one of the main regulators in cellular stress response related to ER [28]. And a previous study has shed light on the relationship of ATF4 with AML, which clarifies that targeting autophagy or ATF4 in patients expressing FLT3 mutations may represent a novel promising and innovative therapeutic strategy for AML [29]. This study provides the possibility that ATF4 not only mediates autophagy in FLT3-mutated AML patients, but also plays a key role in cell proliferation and differentiation mediated by MAPK pathway. The characteristics of elderly AML patients with low ATF4 level are different from the young patients. As more and more evidences show that the identification of new AML biomarkers contributes to a better understanding of the molecular basis of the disease, we hope that ATF4, as a biomarker and the potential therapeutic target, will be significantly useful in diagnosis and prognosis for elderly AML patients, as well as the possibility of indicating new therapeutic strategies [13, 30].

## Supplementary Information

**Additional file 1.** Quality control details of platelet RNA-seq.

**Additional file 2.** Differential transcript levels of genes in platelets between young and elderly AML patients.

**Additional file 3.** KEGG pathway enrichment analysis results.

**Additional file 4.** GO enrichment analysis results of genes with downward transcript levels.

**Additional file 5.** GSEA enrichment analysis results.

## Data Availability

The datasets used and analyzed during the current study are available in the public database https://www.ncbi.nlm.nih.gov/bioproject/PRJNA722042. Accession number: PRJNA722042. Other materials are available from the corresponding author on reasonable request.
